# Supplementary material for: A model for super El Niños
Source: Nat Commun. 2018 Jun 28;9:2528. doi: 10.1038/s41467-018-04803-7 (PMC6023905; doi:10.1038/s41467-018-04803-7)
Supplement: Supplementary file 1 — Supplementary Information [file 41467_2018_4803_MOESM1_ESM.pdf]

Supplementary Information

A model for super El Niños

Saji N. Hameed, Dachao Jin and Vishnu Thilakan

Supplementary Information includes:

- Supplementary Figures 1-24 2–25
- Supplementary References 65-67 26

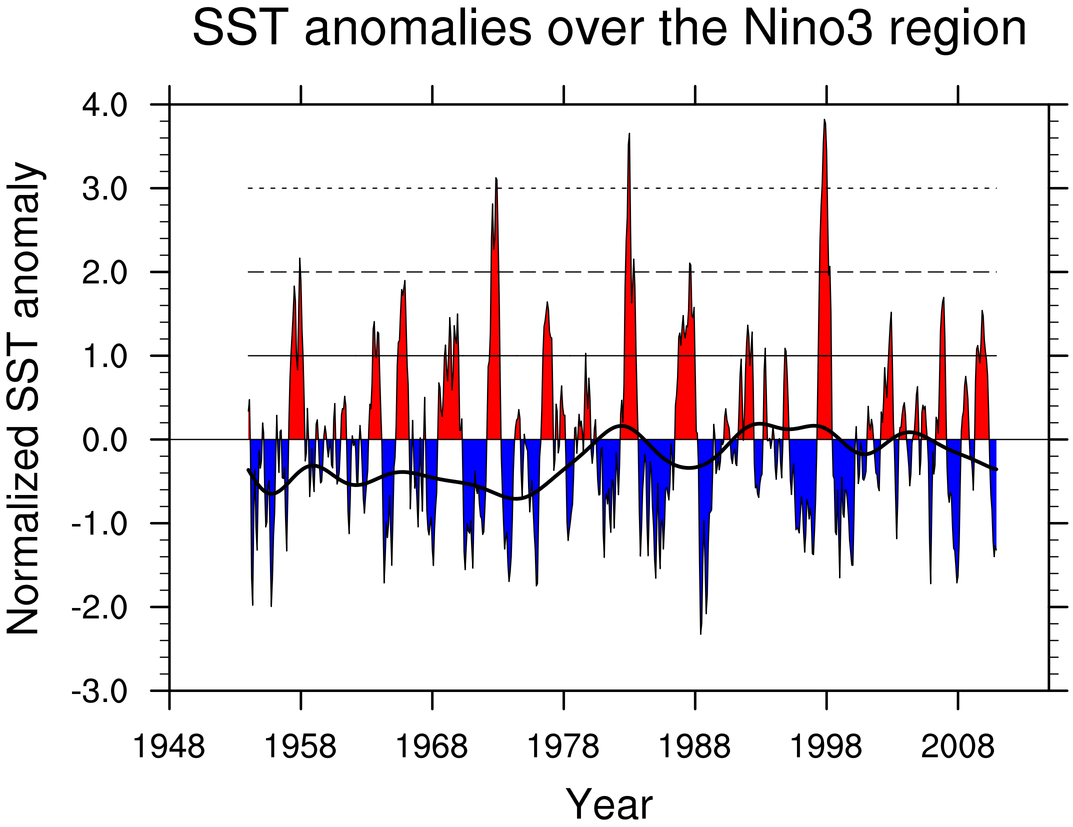

Supplementary Figure 1. Amplitude of filtered monthly SST anomalies over the Nino3 region from 1955 to 2010.
To obtain interannual anomalies, we removed variations with periodicities of 7 years or more from the raw anomalies (mean data minus climatology): the low-frequency (decadal) component (thick solid nine) was estimated by applying a Lanczos filter (low-pass with 169 weights) on monthly anomalies from 1948 to 2017 (data source: HadSST3[58]). The first 7 years and last 7 years are not shown, because these are affected by end-effects of the filtering procedure. The data were normalized by using the standard deviation of the interannual anomalies from 1955 to 2010.

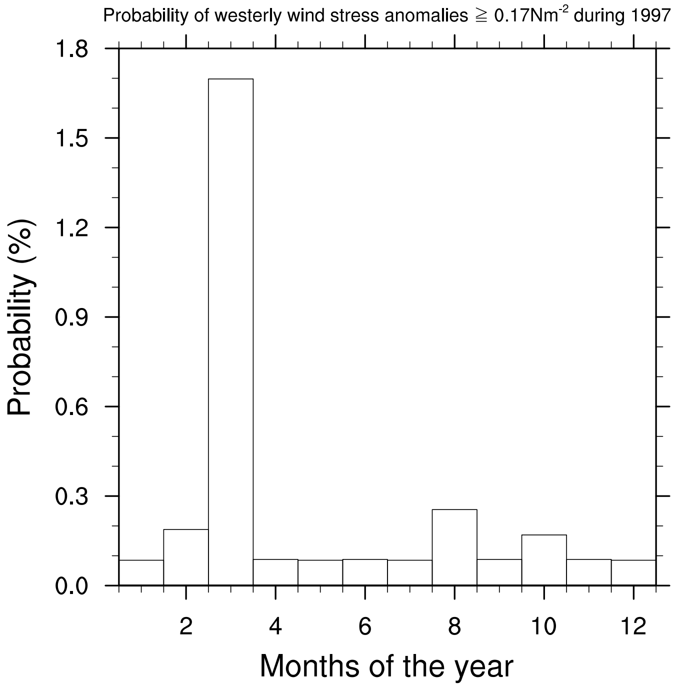

Supplementary Figure 2. Observed frequency of occurrence for extreme westerly wind stress anomalies during the 1997 El Nino.

The frequency of occurrence of westerly wind anomalies (data source: NCEP reanalyses[41]) equal to or exceeding 0.17 Nm^{-2} during each month of 1997 is plotted in the figure, and was calculated as follows. We first distributed daily, equatorially averaged (2.5^{o}S–2.5^{o}N) wind stress anomalies at each of 38 grid points from the western Pacific (120°E) to the central Pacific (190°E) across 25 equally spaced bins. The number of data points where westerly wind anomalies equalled or exceeded 0.17 Nm^{-2} was divided by the total data points in each month, and multiplied by 100 to obtain the probability of occurrence shown on the y-axis. The number of data points where the mentioned threshold was reached is as follows: 1 in January, April–July, September, and November–December; 2 in February and October; 3 in August; 20 in March.

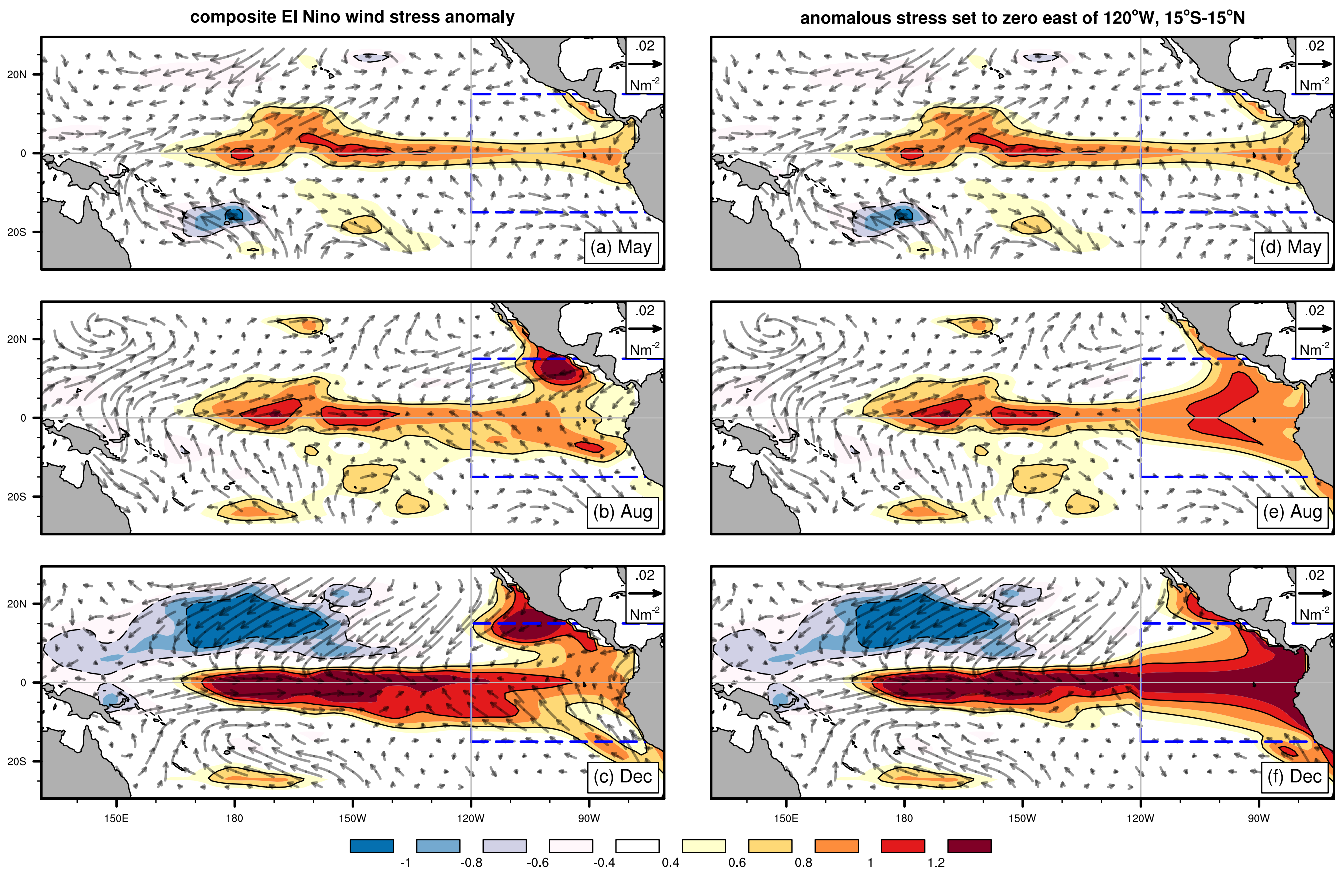

Supplementary Figure 3. The impact of far-eastern Pacific easterly wind stress anomalies on a simulated El Nino. SST anomalies (shaded, units:K) from two ocean model simulations are shown for May (a,d), August (b,e), and December (c,f). (Left): This simulation was forced with composite wind stress anomalies during the 1986,1987,1991, and 2002 El Niños. (Right): In the simulation shown here, the wind forcing was modified by setting the stress anomalies in the far-eastern Pacific (15°S–15°N, 120°W–290°W) to zero, from June to December. This region (bounded by the blue dashed lines in the figures) was chosen, because it experienced easterly wind stress anomalies during the composite El Nino event. The maps in the figure were rendered with the NCAR Command Language software (http://dx.doi.org/10.5065/D6WD3XH5) from the Global Self-consistent, Hierarchical, High-resolution Geography Database (GSHHG). The GSHHG is available online at https://www.ngdc.noaa.gov/mgg/shorelines/gshhs.html).

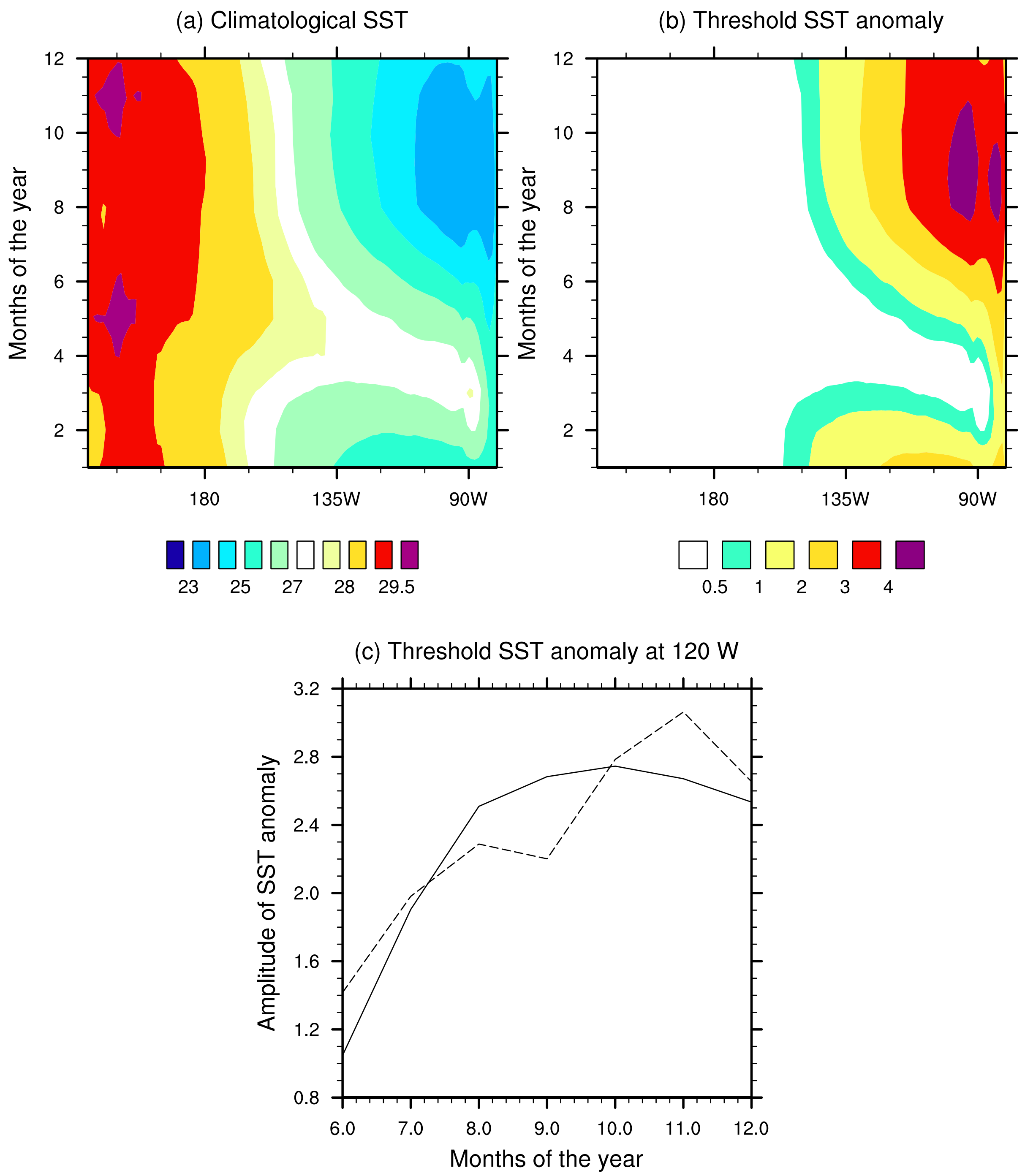

Supplementary Figure 4. The seasonal cycle of SST in the Pacific and its role in limiting the development of anomalous El Nino convection over the cold tongue (a) The seasonal evolution of SST in the equatorial (averaged over 5°S to 5°N) Pacific as a function of longitude and calendar month. (b) shows the minimum SST anomaly that is needed to break the convective threshold[12] of 27.5°C. (c) is the same as (b), but over 120°W from June to December – the solid line shows the smallest warm SST anomaly that can break the convective threshold, while the dashed line is the actual SST anomaly observed during 2015.

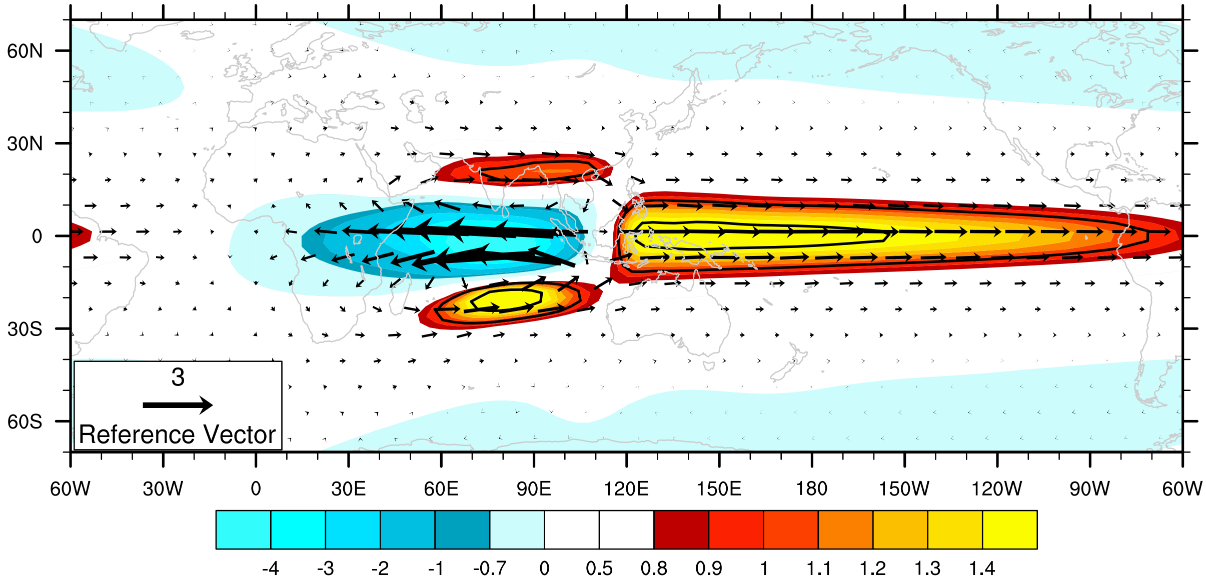

Supplementary Figure 5. The Matsuno-Gill type response to IOD convection The steady state response of winds at 850mb to idealized IOD convection under a resting basic state. The idealized forcing is the same as that in Fig. 5e, corresponding to the developing phase of IOD (August–October). The Kelvin part of the response, found to the east of IOD convection, extends over the entire equatorial Pacific. Under a realistic basic state, the response is substantially modified (Fig. 5e). The maps in the figure were rendered with the NCAR Command Language software (`http://dx.doi.org/10.5065/D6WD3XH5`) from the Global Self-consistent, Hierarchical, High-resolution Geography Database (GSHHG). The GSHHG is available online at `https://www.ngdc.noaa.gov/mgg/shorelines/gshhs.html`).

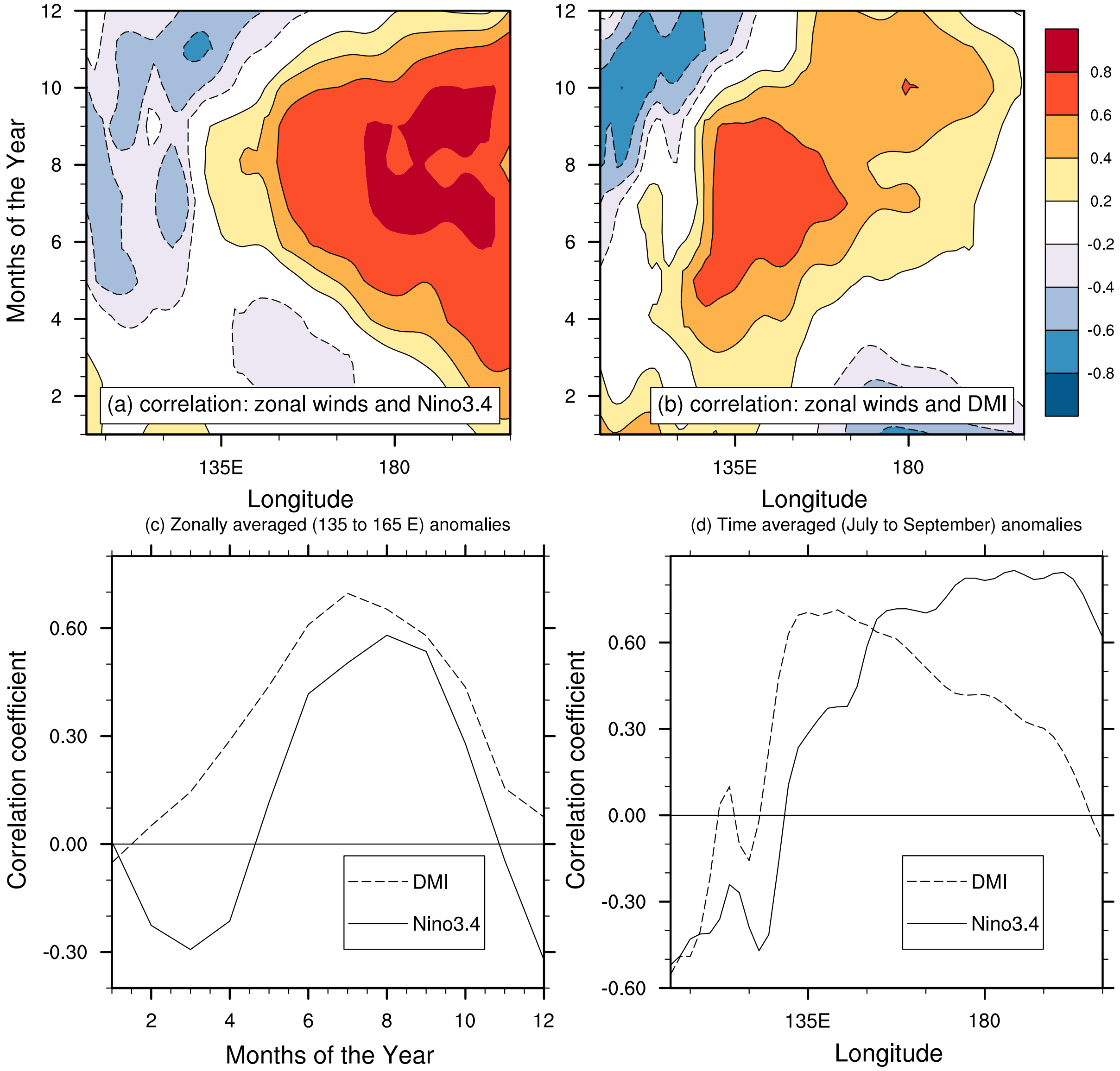

Supplementary Figure 6. The correlation of western Pacific zonal surface wind anomalies with Dipole Mode Index (DMI) and Nino3.4. We correlated equatorially averaged (2.5°S–2.5°N) monthly anomalies from 1958 to 2015, separately, with DMI and Nino3.4 indices – the latter indices were averaged from September to November (the peak season of IOD development). In panels (a,b), the seasonal variation of the correlation over the western and central Pacific is shown: from boreal summer, IOD's influence is clear at the far-western Pacific; by boreal fall significant correlations extend up to the dateline. The amplitude of the correlations over the region 135°–156°E is shown in (c): here, the correlation of wind anomalies with DMI (0.6) is stronger than that with Nino3.4 (0.5) during boreal summer. The longitudinal extent of IOD's influence is shown in (d) by averaging the winds for July-August-September; the IOD response is seen to peak at 150°E, but is also significant over most of the tropical western Pacific warm pool. Note that the interannual standard deviation of equatorial Pacific zonal winds are strongest at the western to central Pacific between 160°E–160°W (Supplementary Fig. 7). Decadal anomalies longer than 7 years were removed from the monthly anomalies. Data during all months of 1972, 1982, and 1997 were excluded from the analysis. The DMI[27] is the difference of SST anomalies in the western (10°S–10°N; 60°E–80°W) equatorial Indian Ocean and the eastern (10°S–Eq;90°E–110°E) equatorial Indian Ocean.

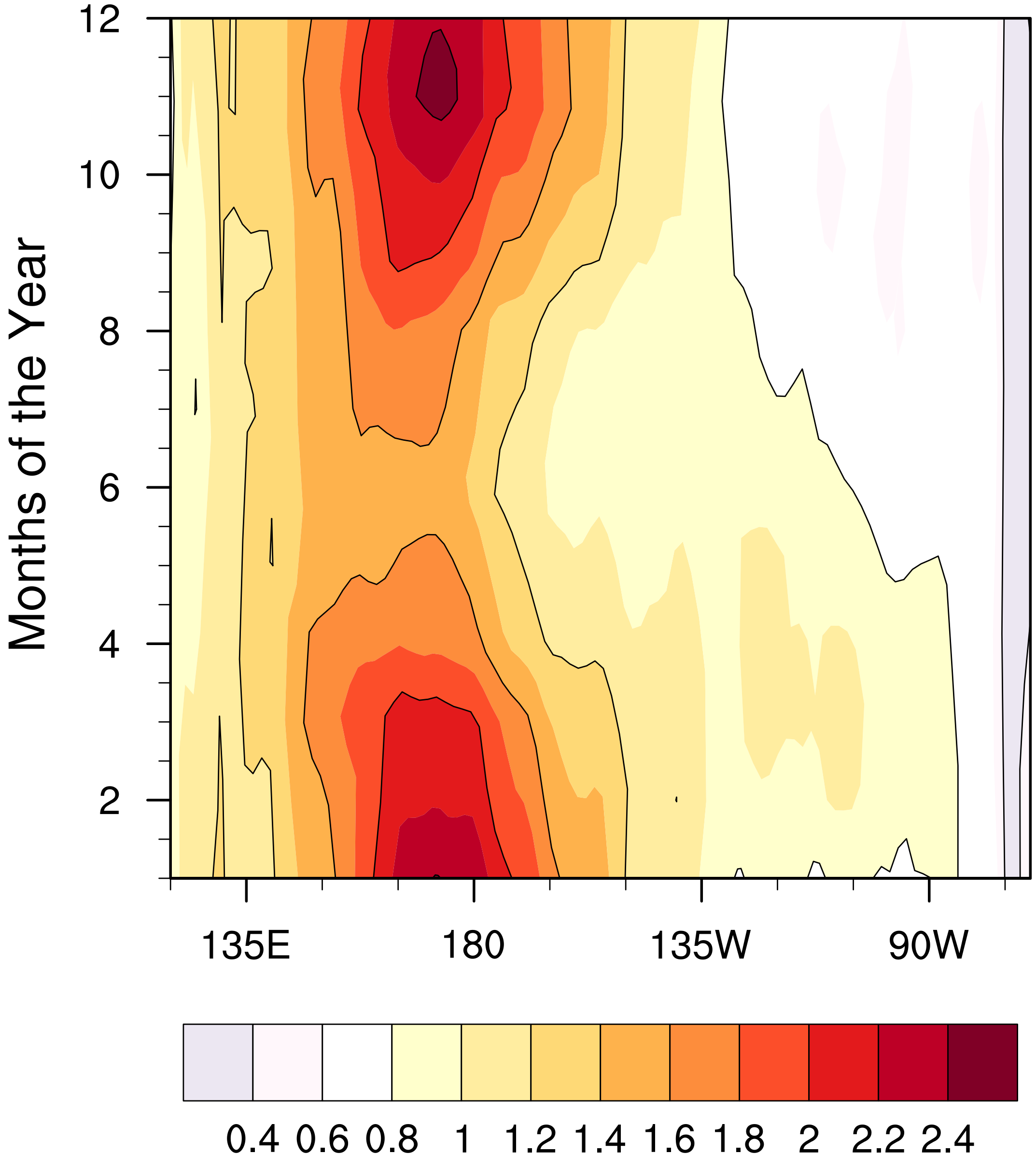

Supplementary Figure 7. Seasonal cycle of the standard deviation of interannual equatorial Pacific zonal wind anomalies. The distribution of interannual standard deviation of zonal wind anomalies, averaged over 2.5°S–2.5°N is shown as a function of longitude and calendar month.

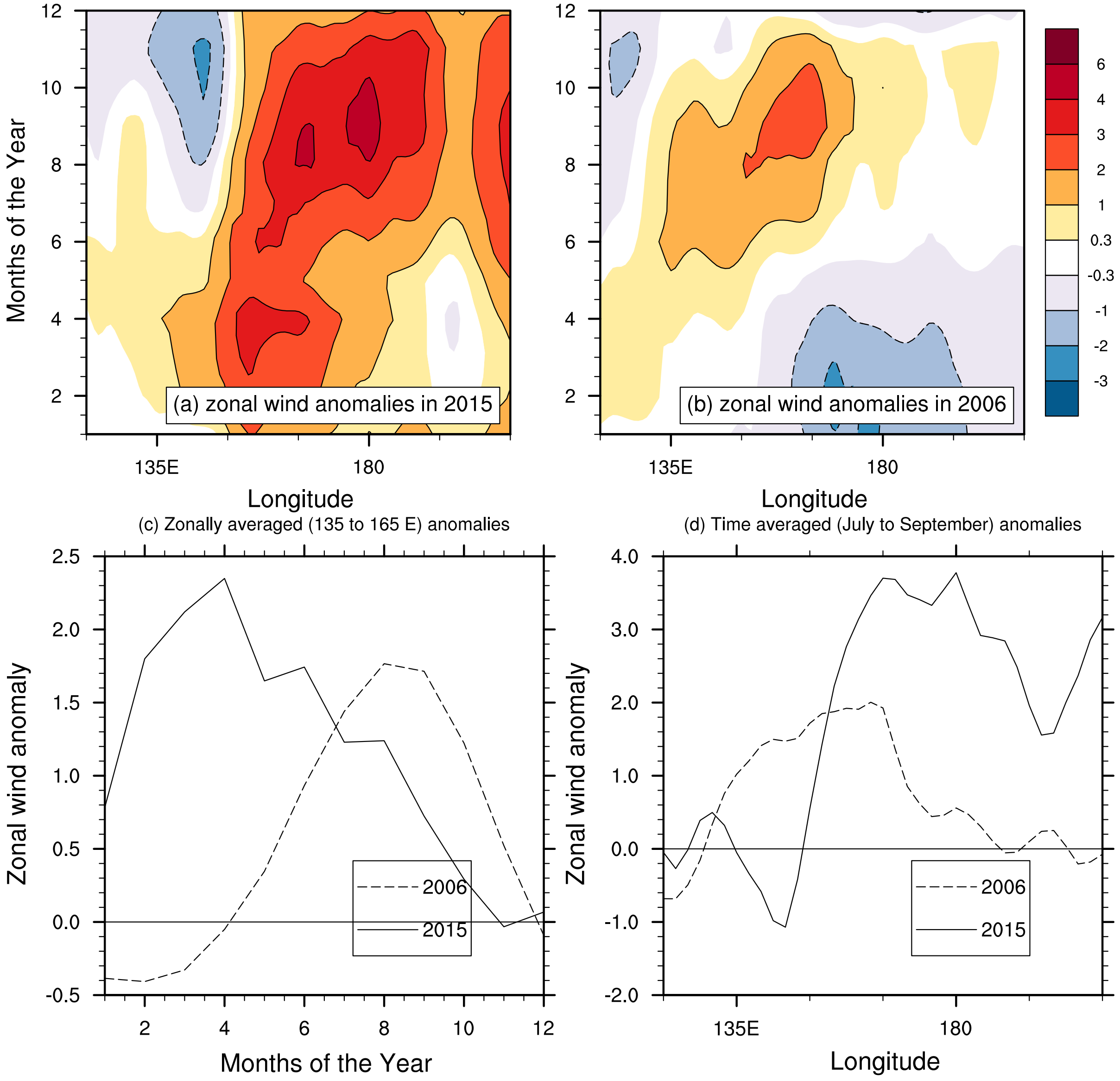

Supplementary Figure 8. The characteristics of IOD-induced Pacific wind anomalies in 2006. Here we compare equatorially averaged (2.5°S–2.5°N) Pacific surface zonal wind anomalies during (a) 2006 with that during (b) 2015. As we argue in the text, western Pacific wind anomalies during the latter half of 2006 can be largely associated with IOD. From June 2015, zonal wind anomalies are weakly positive between 135°E–150°E, and they rapidly change sign and become negative from July onwards. As we estimated from the long term observational record, IOD induces significant westerly wind anomalies over this region (Supplementary Fig. 6b). The structure of IOD-induced winds over the Pacific in 2006 is consistent with this estimate. Panel (c) shows that the relative amplitude of IOD-induced wind anomalies over the far-western Pacific (135°E–165°E) in 2006 is comparable or exceeds that associated with El Nino during 2015. The longitudinal extent of IOD's influence into the Pacific in the late summer of 2006 is demonstrated by averaging the winds for July-August-September (d). The influence of IOD may be estimated to be significant over most of the western Pacific by comparing these wind anomalies during 2006 to that during 2015.

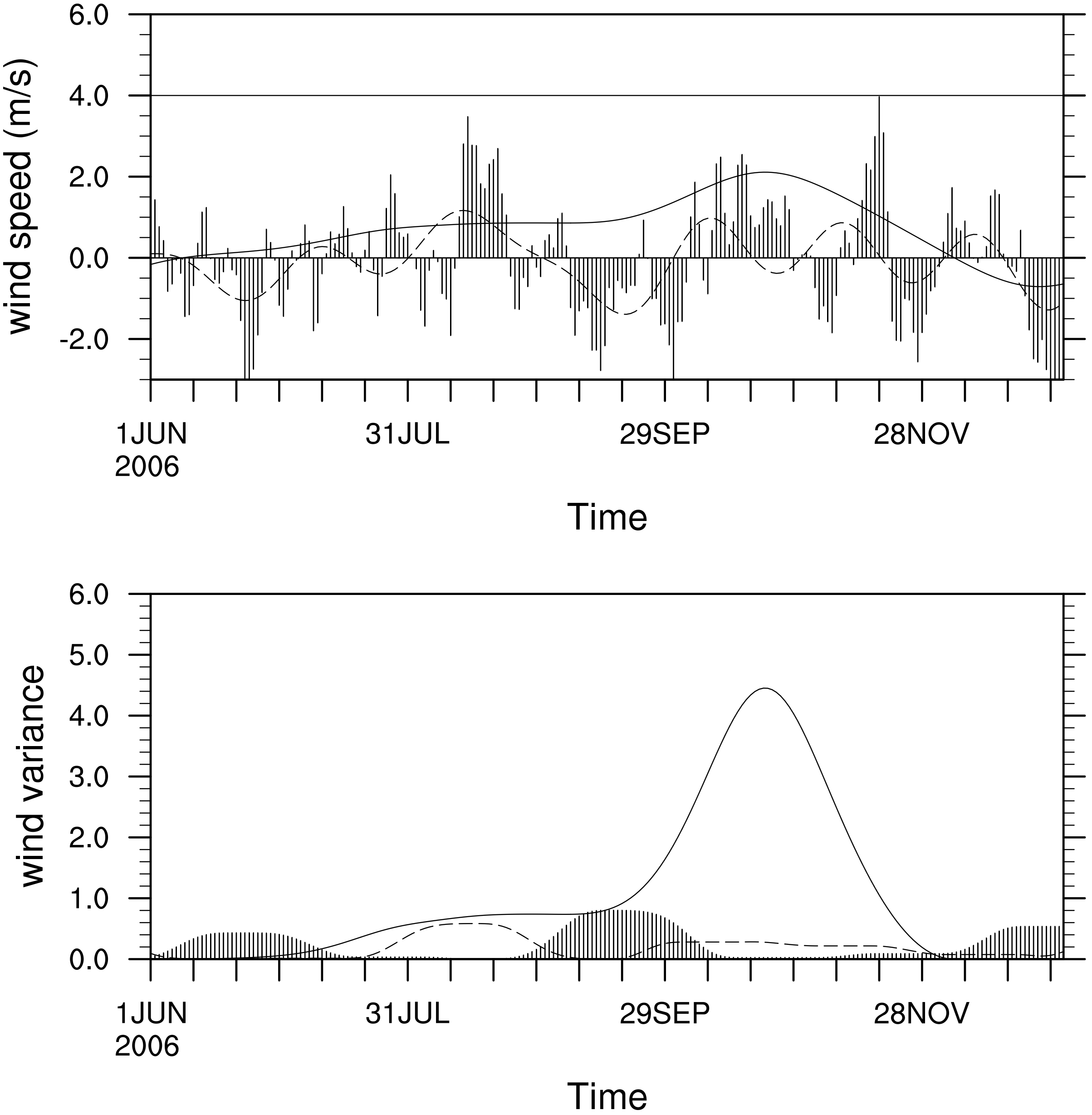

Supplementary Figure 9. The relative strength of interannual and intraseasonal variations in western Pacific wind anomalies during 2006. (Top): Daily zonal wind anomalies in the equatorial western Pacific (5°S–5°N;160°E to 170°W) during the latter half of 2006 are separated into interannual (solid) and intraseasonal (dashed) wind anomalies. The bars show the difference between total daily wind anomalies and the interannual wind anomalies - these include the intraseasonal component. The threshold wind speed used in the WWB scheme is shown as the horizontal solid line. (Bottom): The wind variance from the westerly phase of the intraseasonal anomalies(dashed), easterly phase of the intraseasonal anomalies(bars), and interannual anomalies (solid) were calculated using a moving-window. A window width of 31 days was used (each tick mark is roughly 5 days apart). Elevated westerly intraseasonal activity is seen during early August, when it matches the variance of interannual winds. Easterly wind phases of the intraseasonal variations are strong during three episodes: one around the middle of June, when intraseasonal variance exceeds interannual variance; one at the end of September is of similar amplitude as interannual variance; a third episode is in the middle of December. Overall, intraseasonal variance is weaker than interannual variance, during IOD's development phase, in particular from the middle of August.

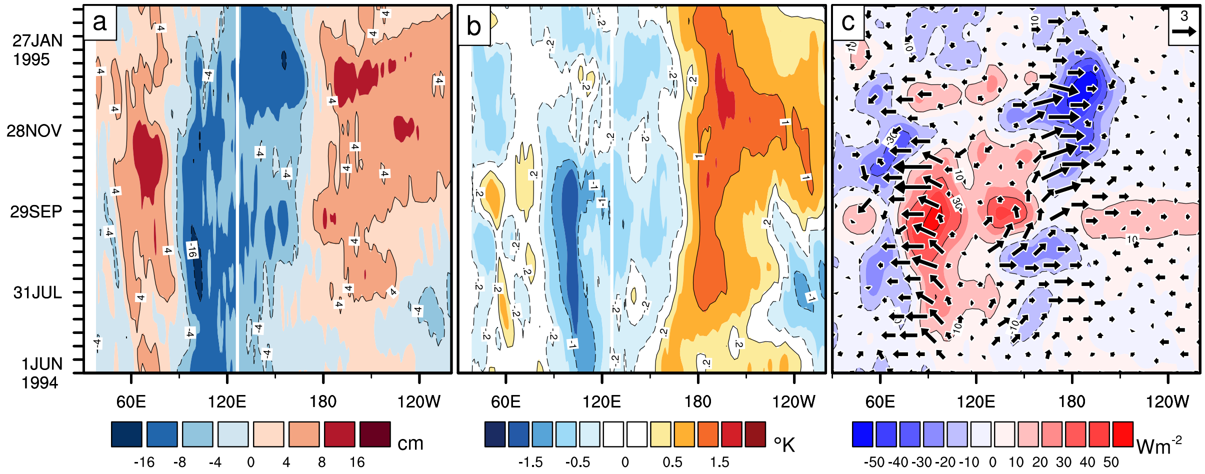

Supplementary Figure 10. Evolution of Indo Pacific climate anomalies during the strong 1994 IOD. The top panels show time-longitude evolution of (a) SSH, (b) SST, (c) OLR and surface zonal wind anomalies (vector, units:ms^{-1}). The onset of the 1994 IOD event started earlier, with a pronounced cooling of the eastern equatorial Indian Ocean in April/May[65;66]. It is to be noted that the El Nino-like SST anomalies over the Pacific during this event was not anticipated, as reflected in the following statement by Kerr (1994, p. 1941)[52]: " the El Nino now developing [December 1994] came as something of a surprise...It is the third in four years, and El Nino forecasts, including that of the NWS's coupled model, didn't see it coming until late summer[1994]. "

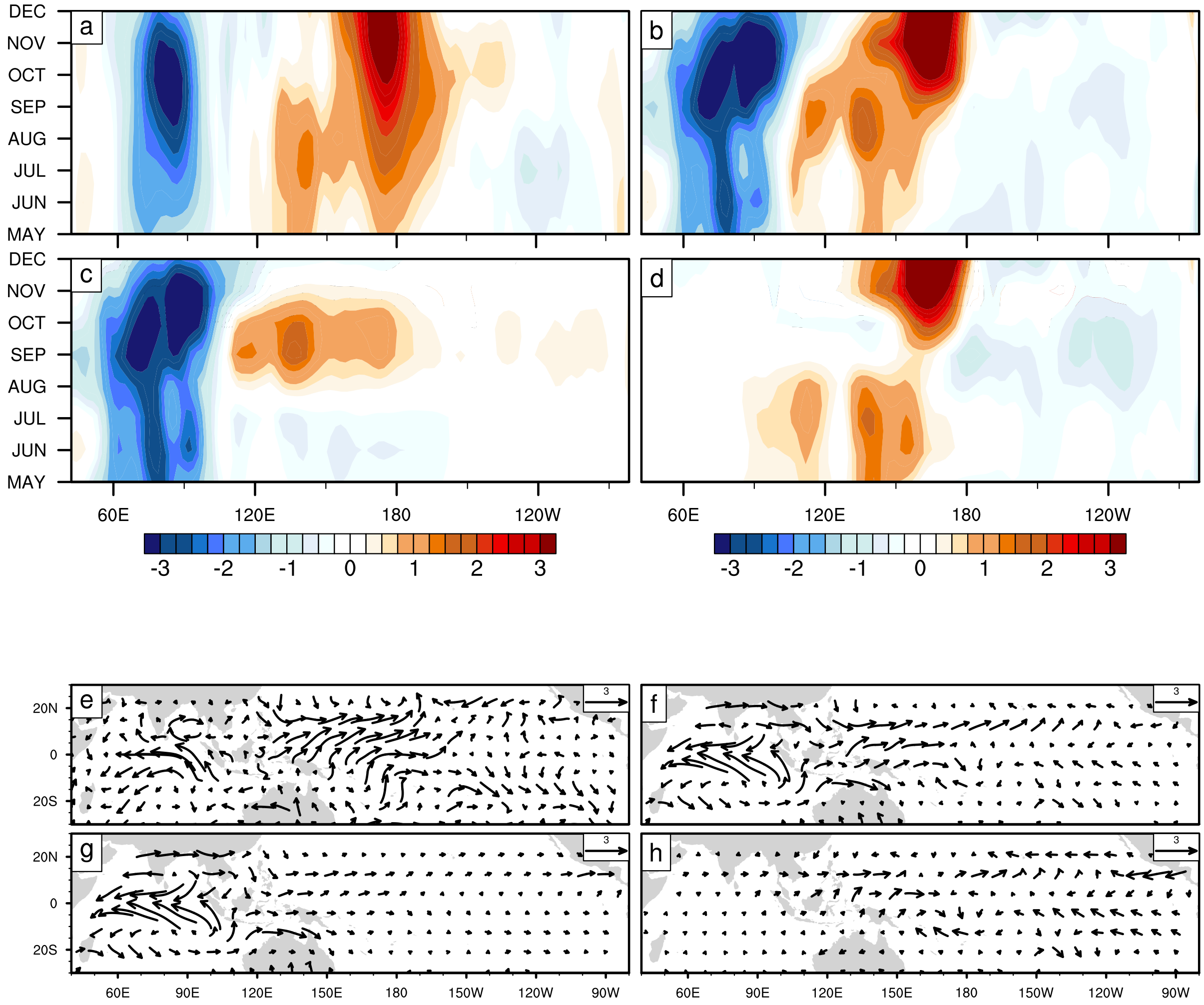

Supplementary Figure 11. Simulation of monthly tropical surface wind anomalies during 1994. The four top panels show longitude-time evolution of monthly equatorial zonal wind anomalies from observations (a) and numerical experiments (b,c,d). In the control experiment shown in (b), the atmospheric model was forced with diabatic heating anomalies over the tropics covering the Indian and Pacific Oceans. In a sensitivity experiment, the forcing was prescribed only over the Indian (c), and in another, the forcing was prescribed only over the Pacific Ocean (d). The four bottom panels show surface wind anomalies, averaged between July and October, for the observations (e) and the numerical experiments (f,g,h); here, (f) is the control experiment, and (g) and (h) are the counterparts of panels (c) and (d) above. Comparing the surface wind anomalies from (g) against that from (f), we estimated that the Indian Ocean forcing alone accounted for for 70% of the simulated zonal wind anomalies till September. After this time, convection locally develops over the warm Pacific SSTA forced by IOD. The surface winds that develop as a local response to the Pacific anomalies dominate after the IOD peak phase. However, these are associated with significant easterly wind anomalies over the eastern Pacific (d,h). All the data were smoothed with a 3-month running mean. The maps in the figure were rendered with the NCAR Command Language software (`http://dx.doi.org/10.5065/D6WD3XH5`) from the Global Self-consistent, Hierarchical, High-resolution Geography Database (GSHHG). The GSHHG is available online at `https://www.ngdc.noaa.gov/mgg/shorelines/gshhs.html`).

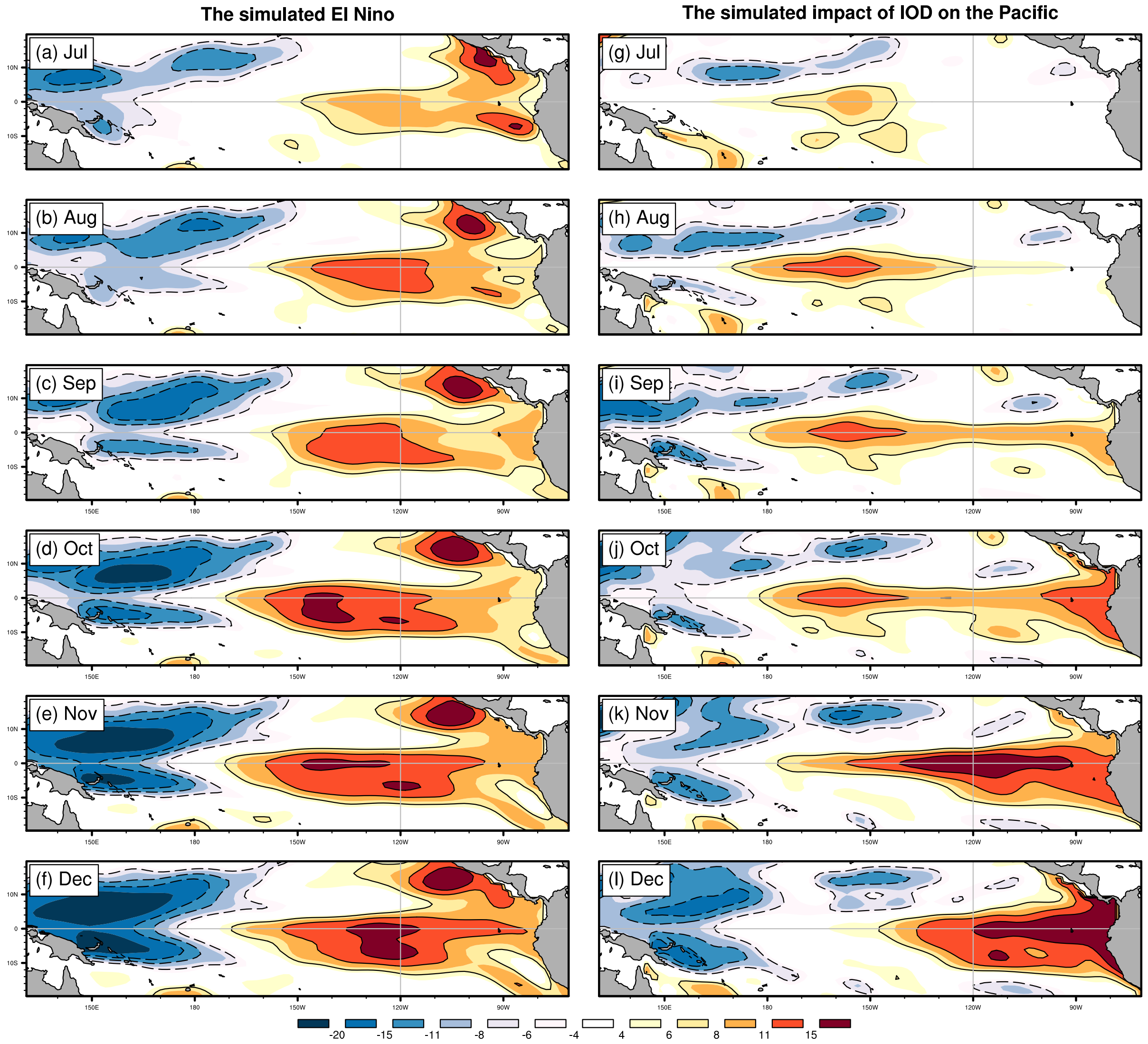

Supplementary Figure 12. The simulated impact of IOD on Pacific thermocline anomalies is compared to the anomalies in a simulated El Nino. (a–f): Evolution of thermocline anomalies during a simulated El Nino. The event was simulated by driving the ocean model with a composite of observed El Nino wind stress anomalies in 1986, 1987, 1991 and 2002. (g–l): Evolution of thermocline anomalies describing the simulated impact of IOD on the Pacific. To simulate the impact of IOD, the ocean model was driven by a composite of observed Pacific wind stress anomalies during the IOD years of 1994 and 2006. The eastern-ocean intensified structure of anomalies induced by IOD's impact can be contrasted with the eastern-ocean damped structure during the simulated El Nino. The maps in the figure were rendered with the NCAR Command Language software (http://dx.doi.org/10.5065/D6WD3XH5) from the Global Self-consistent, Hierarchical, High-resolution Geography Database (GSHHG). The GSHHG is available online at https://www.ngdc.noaa.gov/mgg/shorelines/gshhs.html).

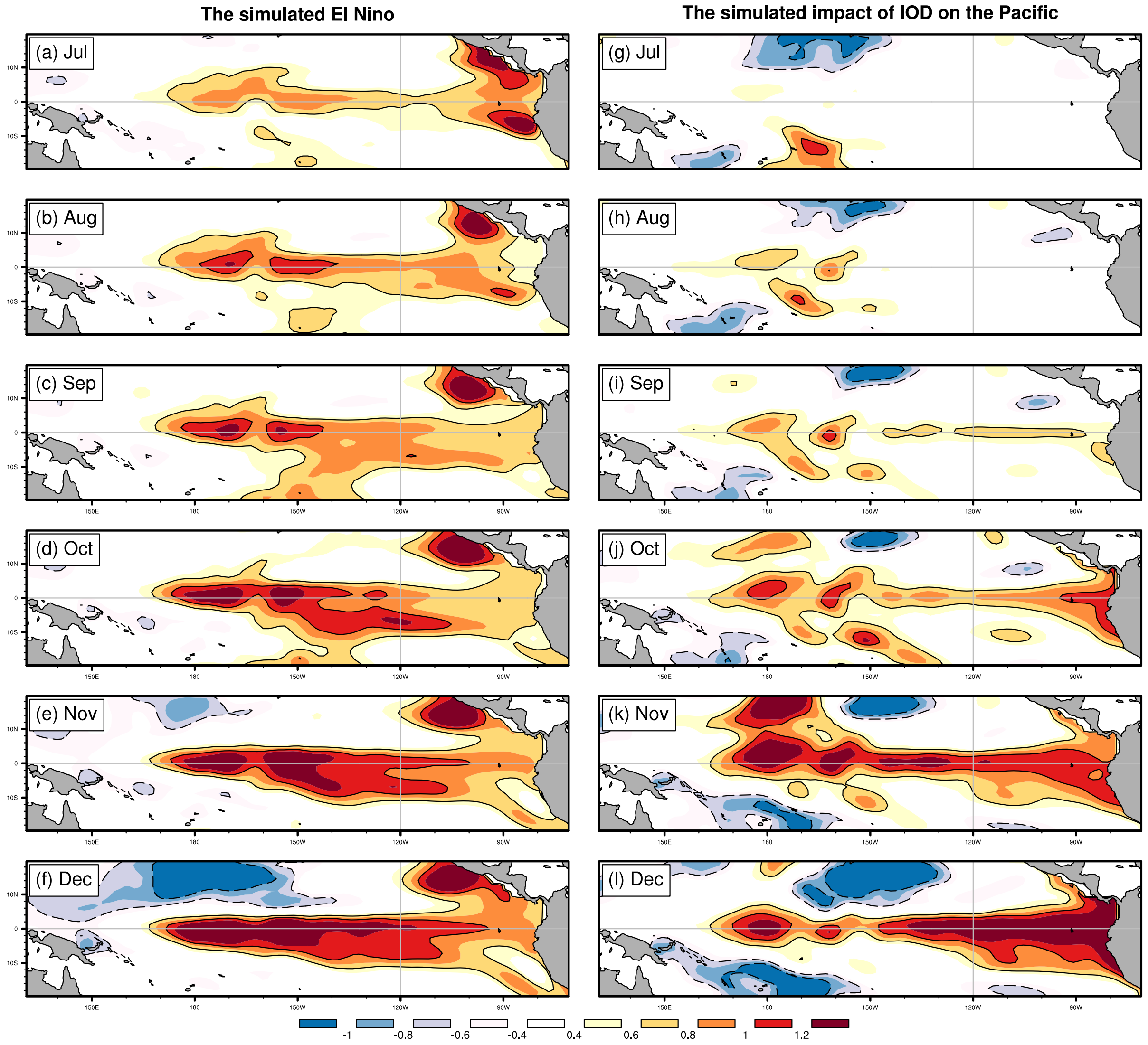

Supplementary Figure 13. The simulated impact of IOD on Pacific SST anomalies is compared to the anomalies in a simulated El Nino. (a–f): Evolution of SST anomalies during a simulated El Nino. The event was simulated by driving the ocean model with a composite of observed El Nino wind stress anomalies in 1986, 1987, 1991 and 2002. (g–l): Evolution of SST anomalies describing the simulated impact of IOD on the Pacific. To simulate the impact of IOD, the ocean model was driven by a composite of observed Pacific wind stress anomalies during the IOD years of 1994 and 2006. The eastern-ocean intensified structure of anomalies induced by IOD's impact can be contrasted with the eastern-ocean damped structure during the simulated El Nino. The maps in the figure were rendered with the NCAR Command Language software (http://dx.doi.org/10.5065/D6WD3XH5) from the Global Self-consistent, Hierarchical, High-resolution Geography Database (GSHHG). The GSHHG is available online at https://www.ngdc.noaa.gov/mgg/shorelines/gshhs.html).

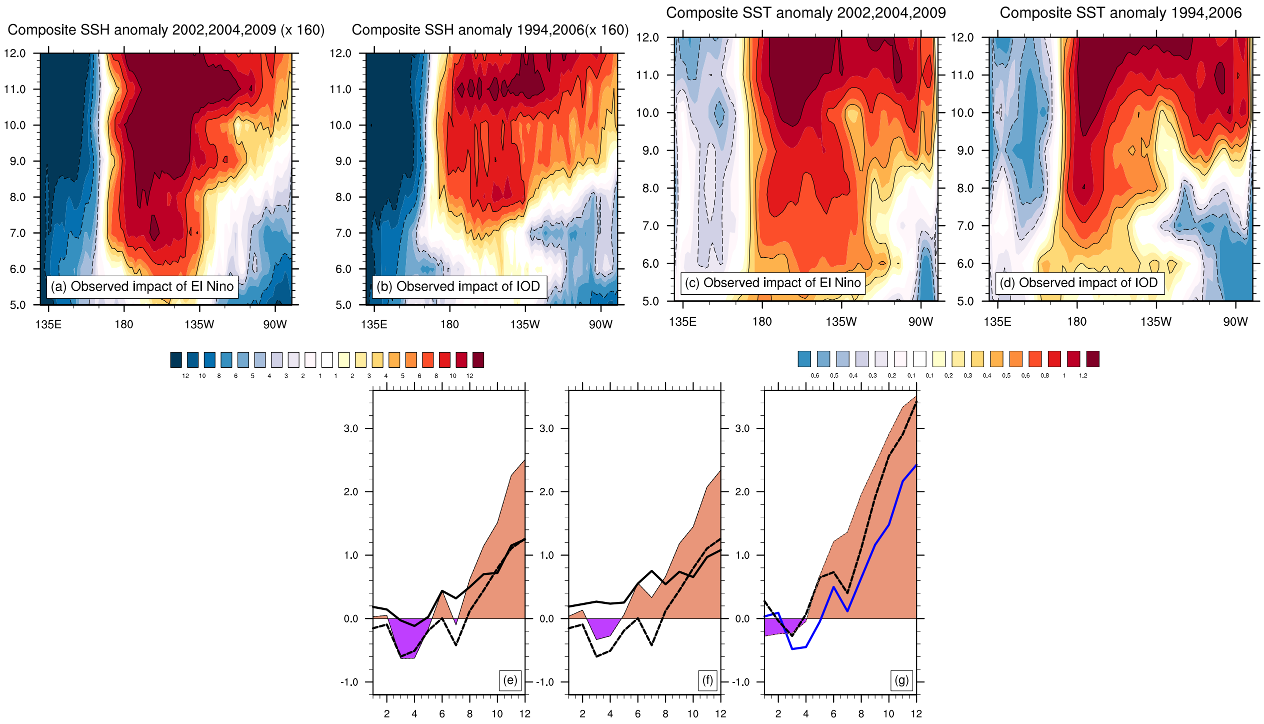

Supplementary Figure 14. Observed Pacific anomalies during El Niño and IOD years. In (a,b,c,d) the evolution of composite equatorial SSH and SST anomalies are shown from May to December. The El Niño composites in (a,c) are from 2002, 2004, and 2009. (*These years were chosen because observed SSH anomalies are available from 1993 only. SST anomalies for these years (e) do not differ significantly from that (f) during other moderately strong El Niño years*). Composite Pacific anomalies during the IOD years of 1994 and 2006 (b,d); dashed lines in (e,f) are Nino3 SST anomalies during these years. The solid lines in (e,f) represent two realizations of a composite El Niño the first from 2002, 2004, and 2009 ; the second from 1986, 1987, and 1991. In panel (g) the solid blue line was constructed by adding the IOD-induced Nino3 anomalies in (e,f) to a composite of Nino3 anomalies from 1986,1987,19971,2002,2004, and 2009. The dashed line show Nino3 anomalies during 1982, while the shaded curve is a composite for the 1982 and 1997 super El Niños.

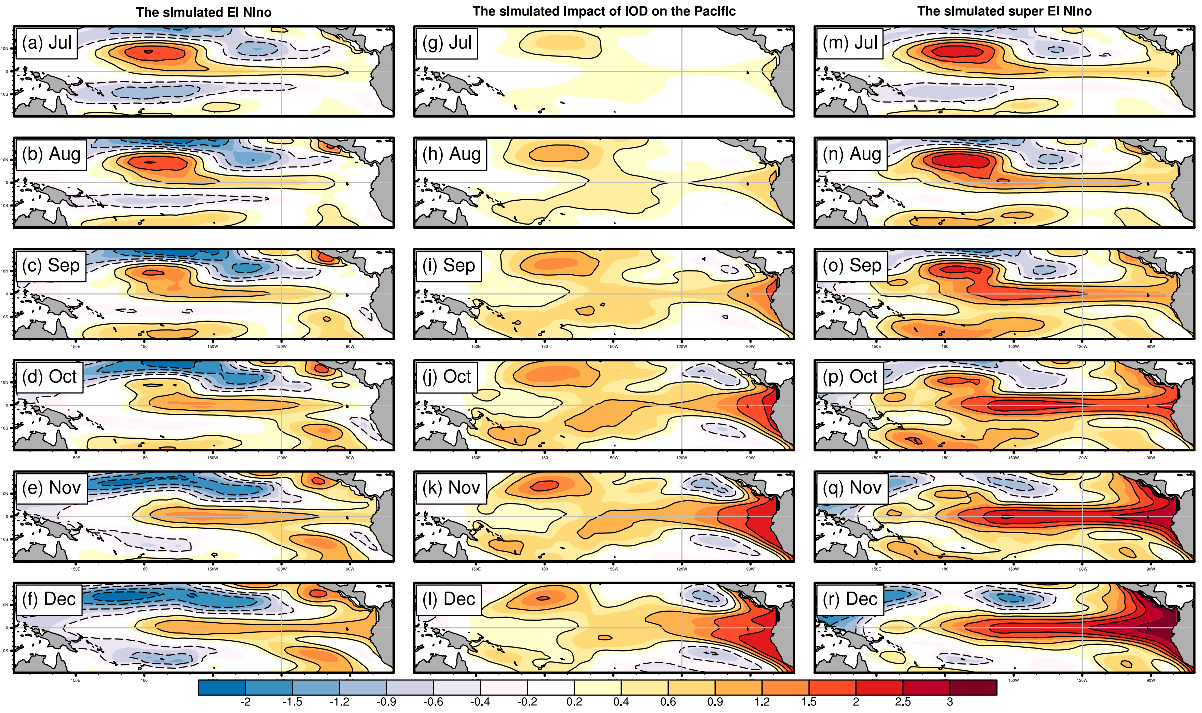

Supplementary Figure 15. Evolution of SST anomalies during a simulated super El Niño. (m–r): In the simulations shown, we drove an ocean model after augmenting Pacific wind anomalies during El Niño with IOD-induced Pacific wind anomalies – the wind anomalies were from atmospheric simulations. The relative contributions of El Niño and IOD to the simulated super El Niño are shown in panels (a–f) and (g–l) respectively. The maps in the figure were rendered with the NCAR Command Language software (`http://dx.doi.org/10.5065/D6WD3XH5`) from the Global Self-consistent, Hierarchical, High-resolution Geography Database (GSHHG). The GSHHG is available online at `https://www.ngdc.noaa.gov/mgg/shorelines/gshhs.html`).

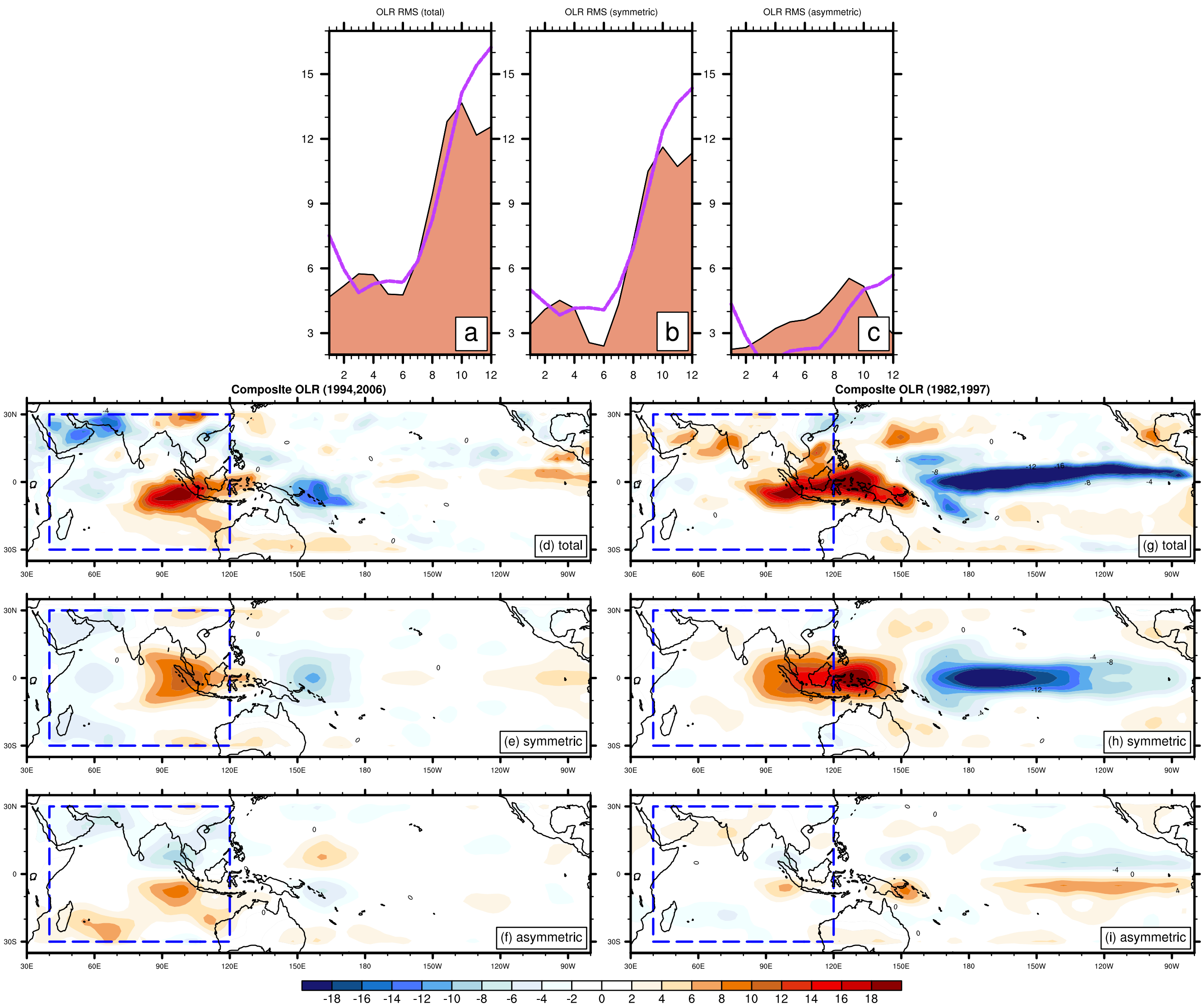

Supplementary Figure 16. Indian Ocean OLR anomalies in IODs that co-occur with El Niños and in IODs that do not co-occur with El Niños. A strong meridional asymmetry characterizes boreal-summer OLR anomalies in IODs that do not co-occur with El Niños. To demonstrate this property, we compare Indian Ocean OLR anomalies from two sets of IOD composites: the first from 1994 and 2006 (d,e,f); the second from 1982 and 1997 (g,h,i). Panel (a) shows root mean amplitude of squared OLR anomaly (olr_{rms}) over the tropical Indian Ocean (20°S–20°N, 40°E–120°E) for the composite OLR anomaly based on 1994 and 2006 (shaded curve) versus that based on 1982 and 1997 (solid line); their symmetric and asymmetric components are shown in (b) and (c), respectively. There are notable differences between the two sets of composites: while the symmetric component (b) is nearly similar in both sets, there are large differences in the asymmetric component (c) during boreal spring and summer. The maps in the figure were rendered with the NCAR Command Language software (`http://dx.doi.org/10.5065/D6WD3XH5`) from the Global Self-consistent, Hierarchical, High-resolution Geography Database (GSHHG). The GSHHG is available online at `https://www.ngdc.noaa.gov/mgg/shorelines/gshhs.html`).

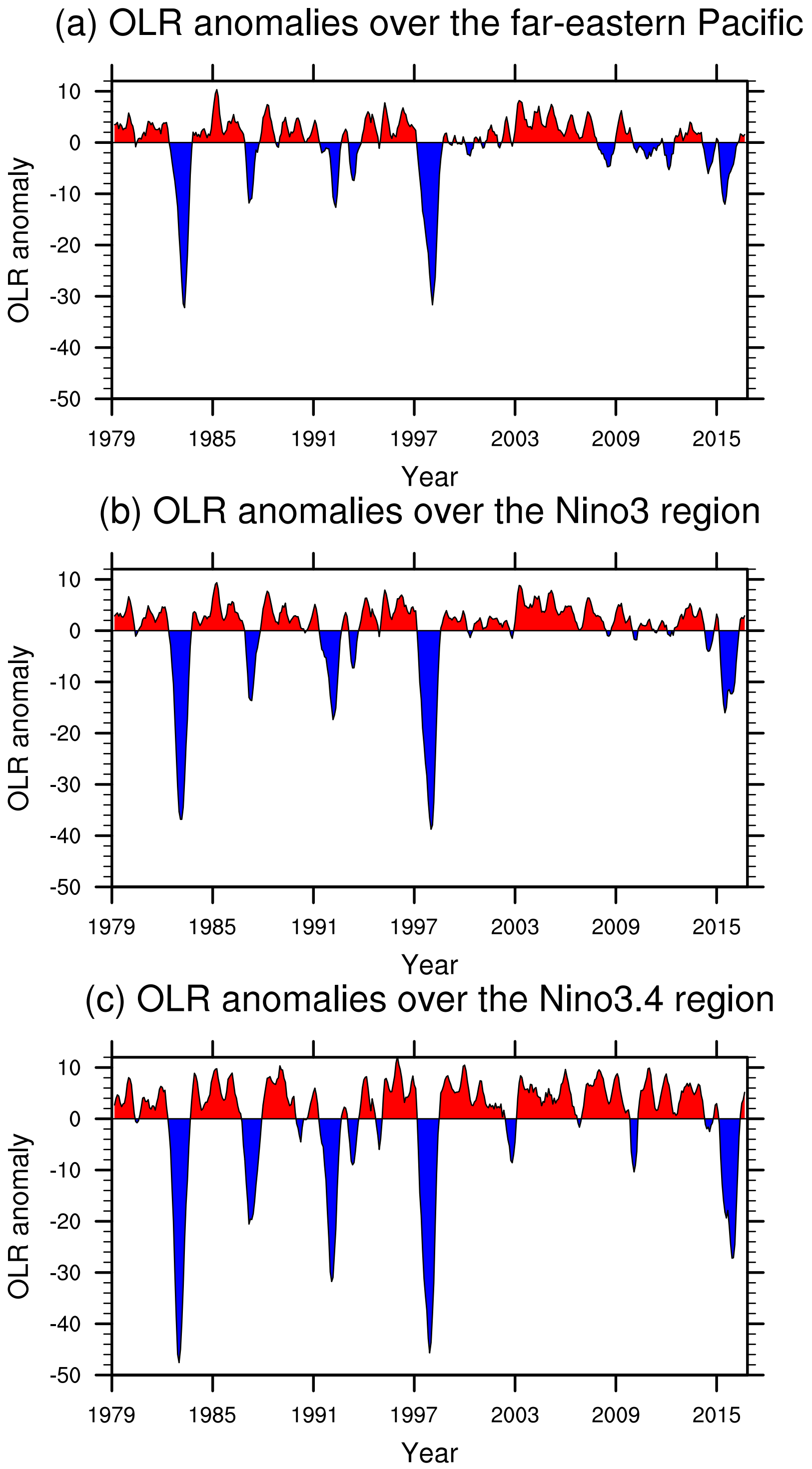

Supplementary Figure 17. Monthly equatorial Pacific outgoing longwave radiation anomalies from 1979 to 2016. (a)–OLR anomalies over the far-eastern (120°–90°W) Pacific. (b)–that over the the Nino3 (150°W–90°W) region. (c)–that over the Nino3.4 (170°W–120°W) region. Equatorial anomalies refer to an average over 5°S–5°N. Except for a 5-month running mean that was applied to smooth the monthly anomalies, no other filters were applied, nor were trends removed from the data.

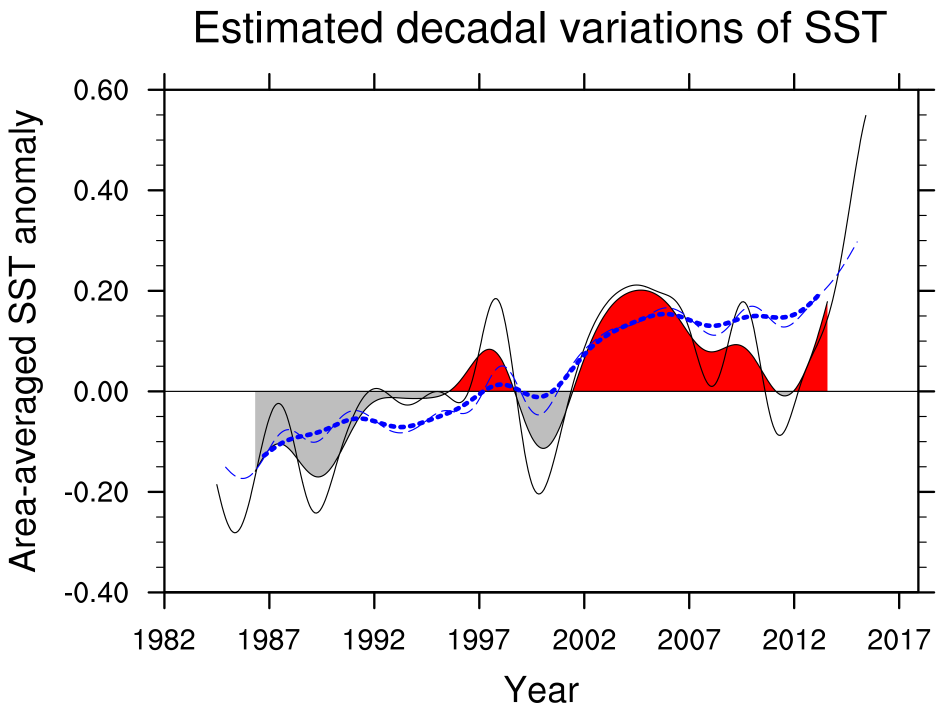

Supplementary Figure 18. Estimated decadal time-scale variations in SST. Decadal anomalies in globally-averaged SST are shown as blue dashed curves: the thick curve was found using a Lanczos filter with 91 weights, the thin curve using a filter with 49 weights. The shaded curve shows the decadal signal in tropical Pacific (30°S–30°N, 140°E–290°E), found with a filter that had 91 weights (the thin black curve is the same metric using 49 weights). Decadal anomalies over the Pacific exhibit a weakening trend from 2004 to 2011, and then a rapid warming after 2012. These decadal trends are reflected on global decadal SST anomalies with a reduced amplitude. *There are disadvantages and advantages to using a large number of weights. On the one hand, a certain number of data points (roughly half the number of filter weights) have to removed from each end of the filtered time series, because of the end-effects of the filter. The number of weights, on the other hand, affect the frequency response of the filter (how much data at each frequency is permitted to remain in the filtered data).* A filter with large number of weights (e.g. 91) is ideal, but does not permit us to estimate the decadal signal during the 2015 El Niño – a 49 weight filter, however, permits us to estimate decadal anomalies until the end of 2015, with an input data spanning Jan 1982 to Dec 2017. This (less than optimal) filter lets in a fraction of interannual anomalies (Supplementary Fig: 19). *However, a comparison with the signal using 91 weights suggests the robustness of the accelerated warming trend in tropical Pacific and global SST since 2012. Further, interannual frequencies are not visible in the signal using 49 weights, neither in global SST nor in tropical Pacific SST: instead, a clear monotonic, accelerated warming trend is evident from 2012 onwards.*

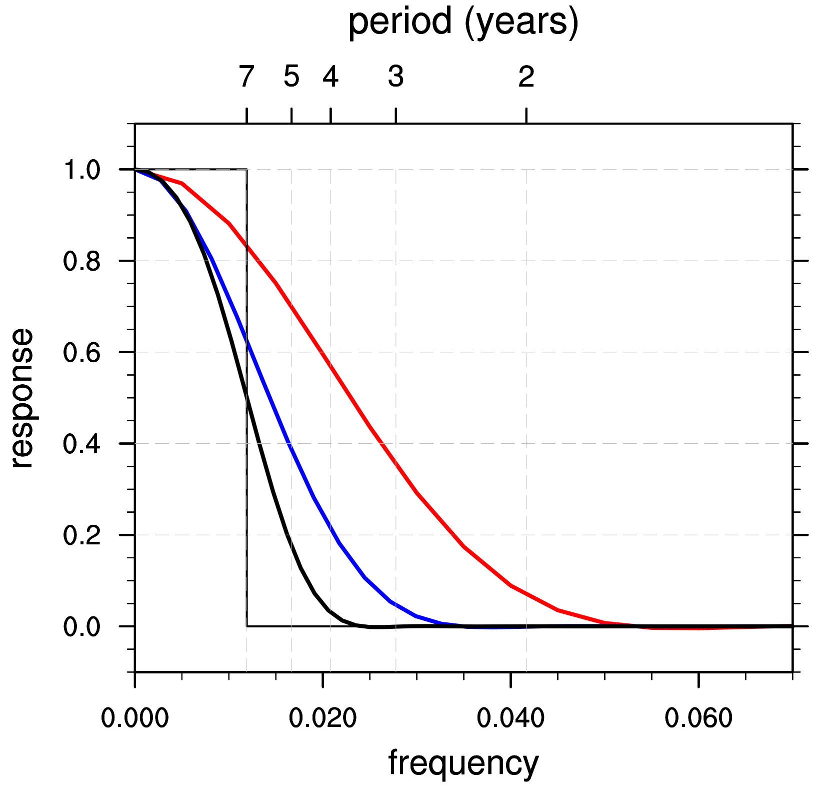

Supplementary Figure 19. The Response function of the Lanczos filters used in this study. The response function shows the fraction of signal that remains after filtering, as a function of frequency. The response function curves shown in red, blue, and black correspond to Lanczos filters that used 49, 91, and 169 weights respectively. Filters with lower weights have a less than optimal response function: the filter with 49 weights lets in a bit of interannual variations, especially those with periodicities around 4 years and longer.

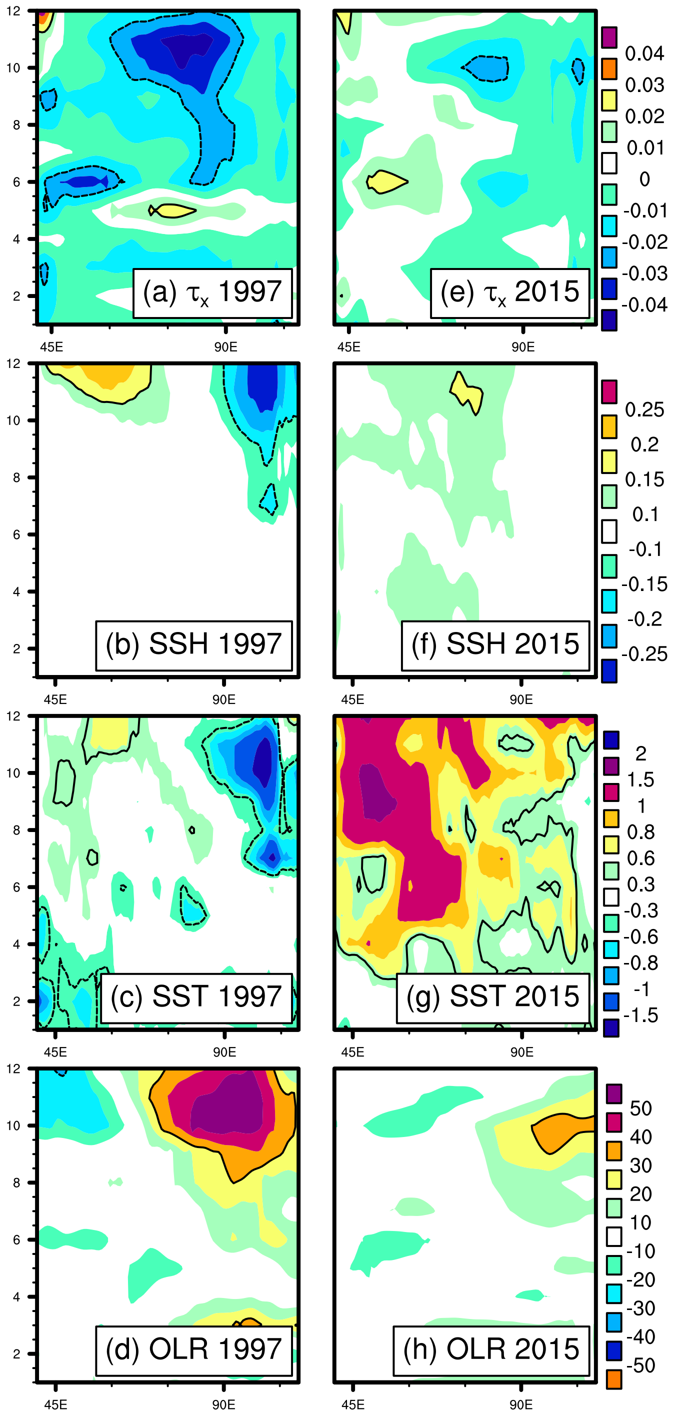

Supplementary Figure 20. Comparing tropical Indian Ocean climate anomalies during two strong El Ninos - 1997 (a–d) and 2015 (e–h). Anomalies of (a) zonal surface wind stress (τ_x), (b) SSH, (c) SST, and (d) OLR during 1997, from January to December ; (e)–(h) are the same as (a)–(d), but for 2015. Zonal surface wind stress anomalies were averaged between 5°S and 5°N. SST, SSH, and OLR anomalies were averaged from 10°S to the Equator, considering their strong equatorial asymmetry during IOD.

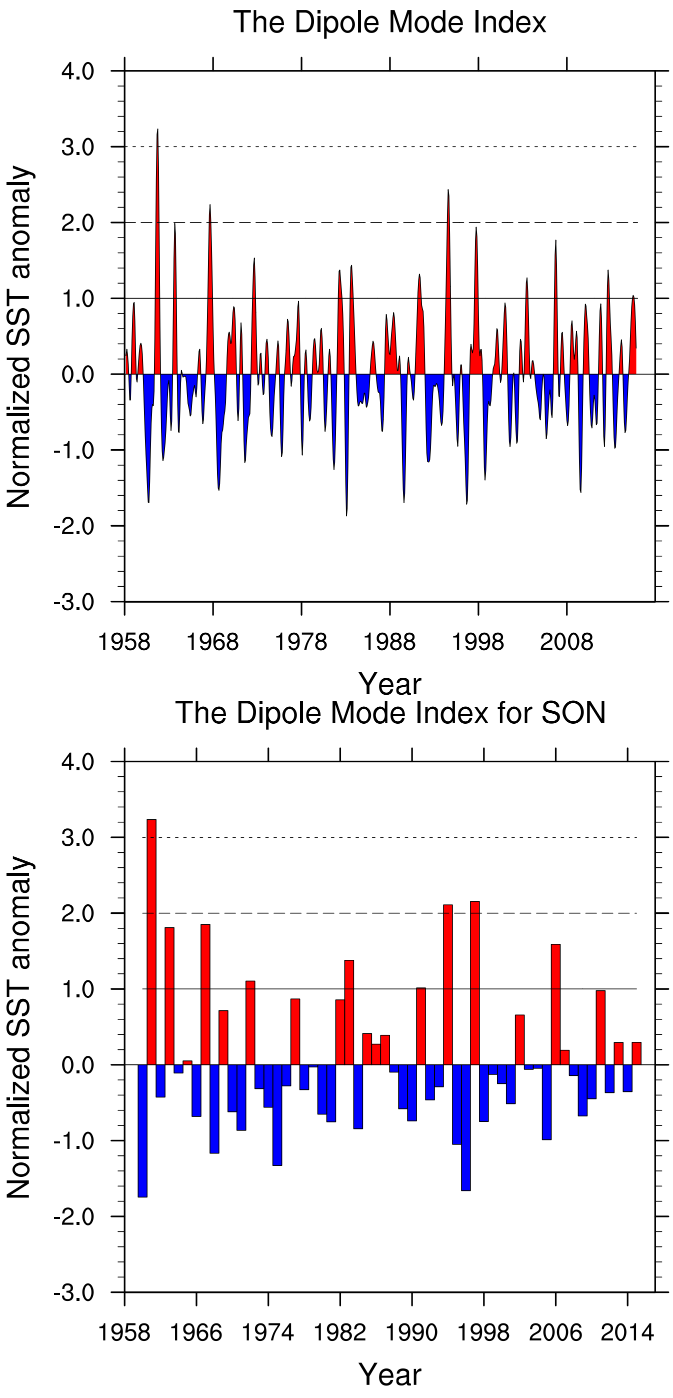

Supplementary Figure 21. Filtered monthly (top) and seasonal (bottom) DMI indices from Jan 1958 to Nov 2015. Decadal variations in SST anomalies were removed prior to computing the index, using a Lanczos filter with 49 weights. ENSO induces basin-scale anomalies on the Indian Ocean - these lag ENSO by 3 to 6 months, and have the same sign as Nino3 SST anomalies; this basin-scale teleconnection of ENSO was estimated using lagged regression, following Saji and Yamagata[27], and removed from the Indian Ocean data, prior to computing the DMI. Note that DMI is elevated from Novemeber 2014 and peaks in July 2015. After July 2015, DMI plunges sharply to zero in October 2015, so that during the boreal fall of 2015, DMI variation is negligibly small ($< 0.2\sigma$). This behaviour of DMI is not representative of an IOD event[26;27].

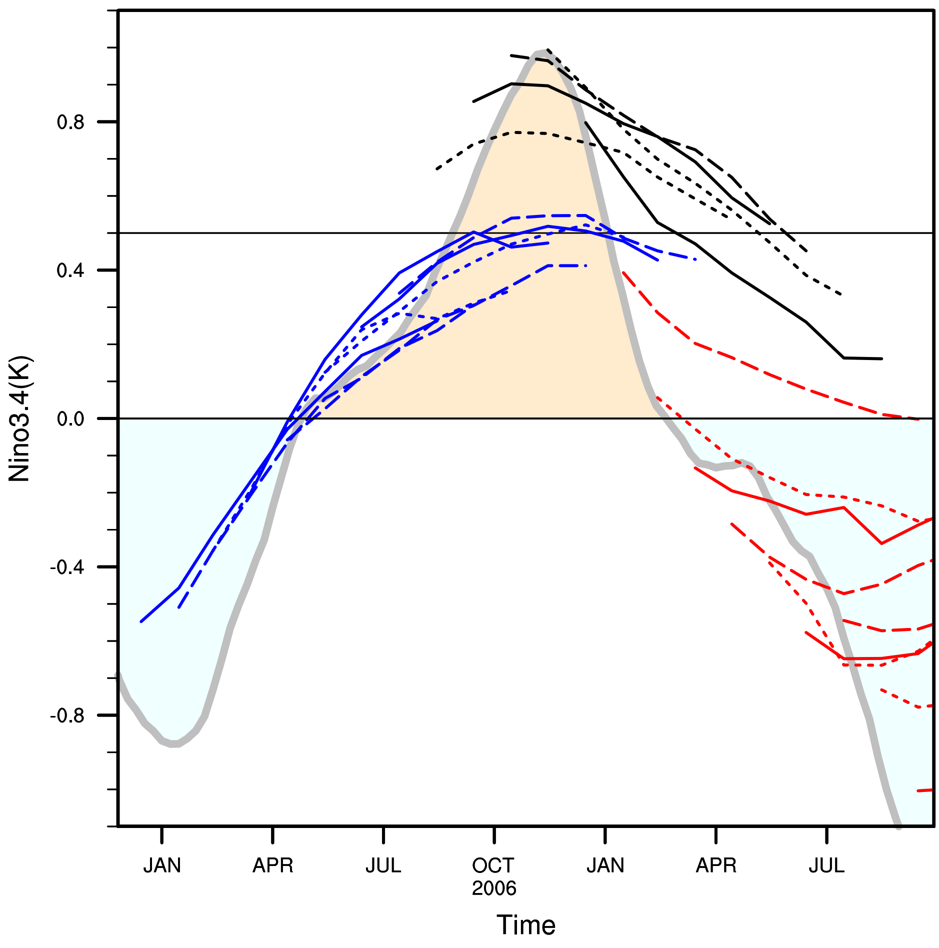

Supplementary Figure 22. Nino3.4 forecasts in 2006. The solid gray curve shows evolution of observed Nino3.4 SST anomalies from January 2006 to December 2007. Each colored curve is a multi-model averaged real-time forecast of Nino3.4 SST anomaly—these are shown for the next nine overlapping 3-month seasons. The first forecast starts a month following the latest observation. The multi-model ensemble forecast was realized by averaging multiple Nino3.4 SST anomaly forecasts from prediction models all over the world. The details of these prediction models can be found from Tippet et al[67] or from `http://iri.columbia.edu/our-expertise/climate/forecasts/enso/current/?enso_tab=enso-sst_table`. The data can be downloaded from `http://iri.columbia.edu/~forecast/ensofcst/Data/` Note that although warm Nino3.4 SST anomalies were correctly predicted, on average the forecasted anomalies were below the 0.5 degree threshold for El Nino. The forecasts also did not capture the peak value of the 2006 event. Further, they failed to predict the rapid termination of the event.

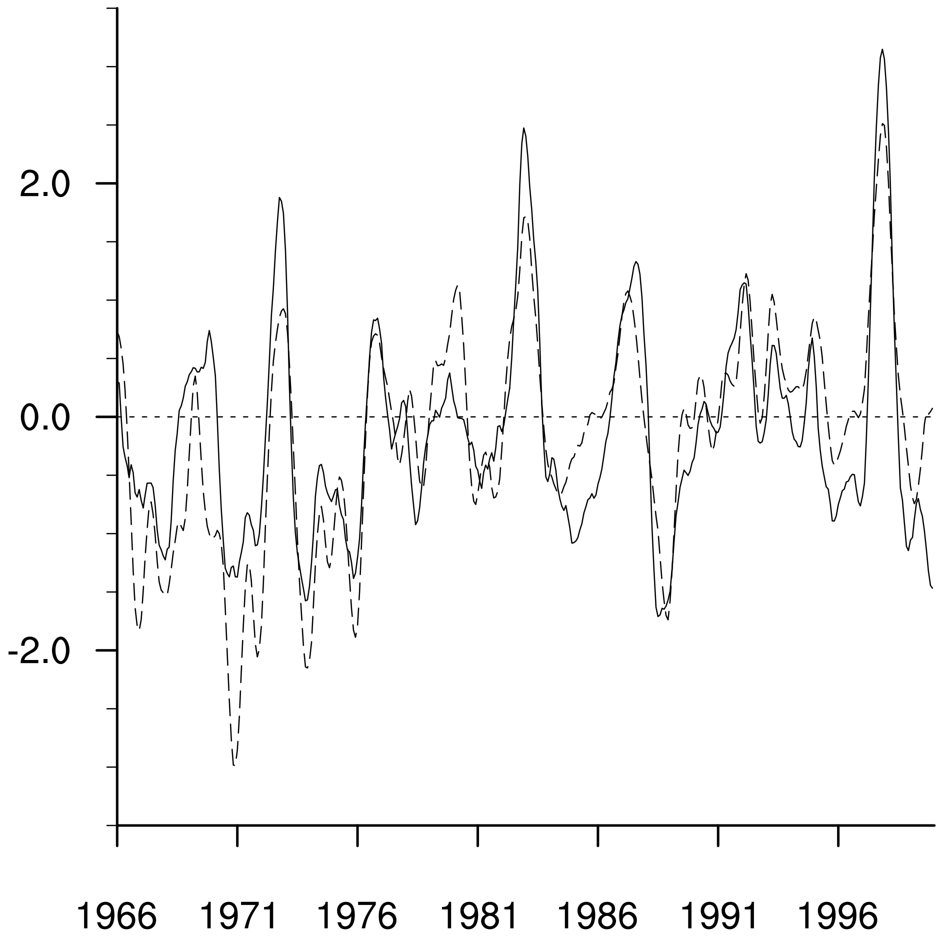

Supplementary Figure 23. Comparison of the Nino3 index from observations and the ocean model. The solid line is the Nino3 index from observed SST over the period Jan 1966 to Dec 1999. The dashed line shows the simulated Nino3 index from the ocean model forced by NCEP wind stress anomalies. The ocean model captures the time evolution of Nino3 anomalies fairly well, but underestimates the amplitude of SST anomalies. The ocean model was originally tuned to the FSU pseudostress anomalies[61]. We did not readjust the model parameters. However when the simulated SST is shown in other figures (not this one), it is multiplied by a factor of 1.5

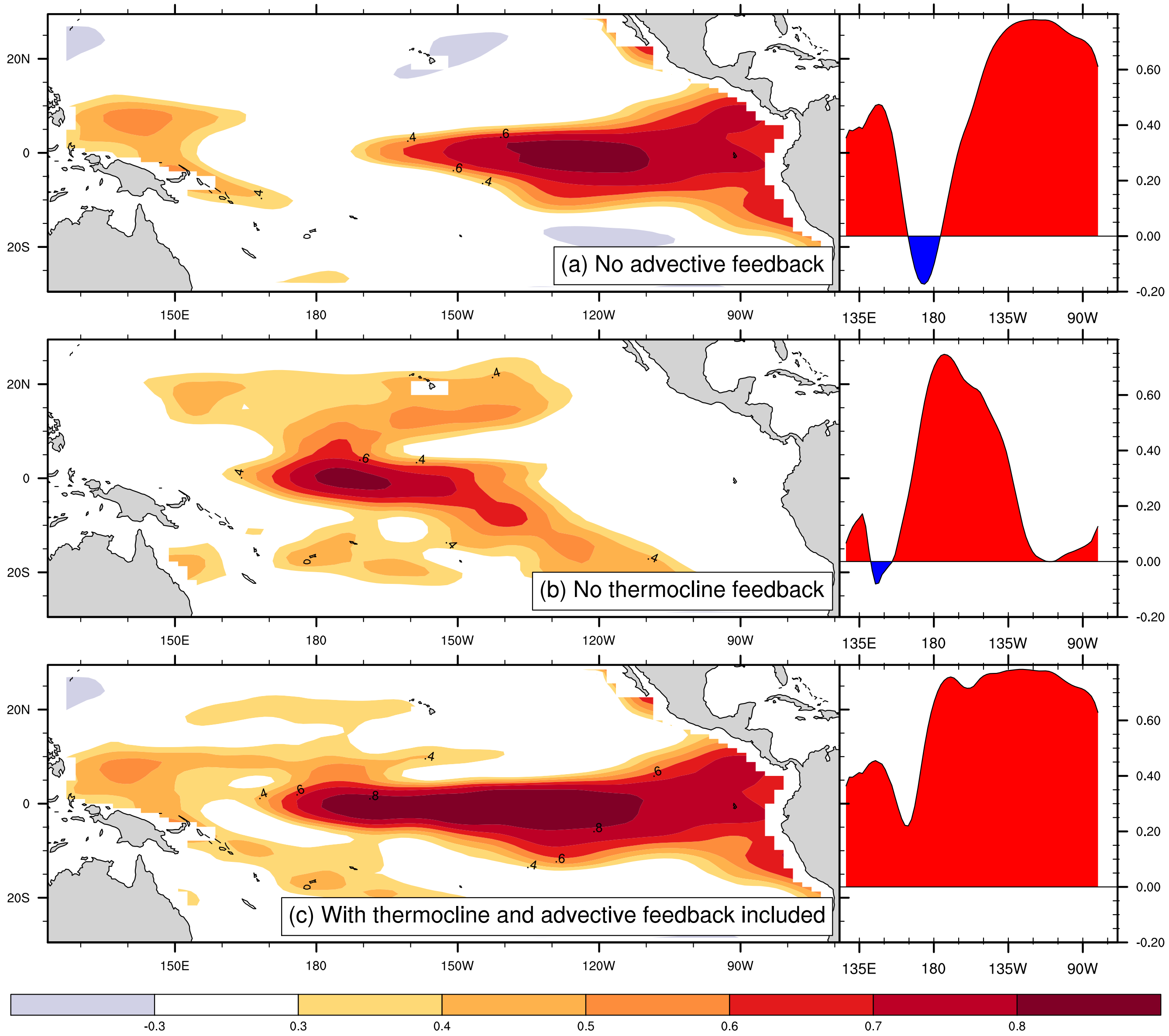

Supplementary Figure 24. Correlation between observed and simulated SST anomalies and their sensitivity to terms in the ocean model's SST equation The bottom panel (c) shows correlation coefficent between SST anomalies simulated by the ocean model and that from observations (OISST[55]) for the period 1979 to 1999. Panels (a) and (b) are the same, except that in (a) the zonal advection feedback was switched off, while in (b) the thermocline feedback was switched off. The line plots, attached to the right of the correlation maps, show the correlation coefficient averaged between 5°S and 5°N. The maps in the figure were rendered with the NCAR Command Language software (http://dx.doi.org/10.5065/D6WD3XH5) from the Global Self-consistent, Hierarchical, High-resolution Geography Database (GSHHG). The GSHHG is available online at https://www.ngdc.noaa.gov/mgg/shorelines/gshhs.html).

Supplementary References

65. Vinayachandran, P. N., Saji, N. H. & Yamagata, Toshio. Response of the equatorial Indian Ocean to an unusual wind event during 1994. *Geophys. Res. Lett.* **260**, 16131616 (1999).

66. Meyers, G. Variation of Indonesian throughflow and the El Niño-southern Oscillation. *J. Geophys. Res. Oceans* **1010**(C5), 1225512263 (1996).

67. Tippett, M. K., Barnston, A. G. & Li, S. Performance of recent multimodel ENSO forecasts. *J. Appl. Meteorol. Climatol.* **510**, 637654 (2012).
